# Memantine Use and Cognitive Decline in Huntington's Disease: An Enroll‐HD Study

**DOI:** 10.1002/mdc3.13763

**Published:** 2023-05-16

**Authors:** Amy C. Ogilvie, Jordan L. Schultz

**Affiliations:** ^1^ Department of Epidemiology The College of Public Health at the University of Iowa Iowa City IA USA; ^2^ Department of Psychiatry The Carver College of Medicine at the University of Iowa Iowa City IA USA; ^3^ Department of Neurology The Carver College of Medicine at the University of Iowa Iowa City IA USA; ^4^ Division of Pharmacy Practice and Sciences The College of Pharmacy at the University of Iowa Iowa City IA USA

**Keywords:** Huntington's disease, cognition, memantine, progression

## Abstract

**Background:**

Memantine is an *N*‐methyl‐*d*‐aspartate (NMDA) receptor antagonist that is used to treat moderate to severe Alzheimer's Dementia (AD) and has been speculated to provide clinical benefits in Huntington's disease (HD).

**Objective:**

To assess the effectiveness of memantine on the trajectory of cognitive decline in individuals with manifest HD.

**Methods:**

Using participants from the Enroll‐HD study, the primary analysis compared trajectories in cognition over a 5‐year period using linear mixed effect models of prevalent and incident memantine users who were propensity‐score‐matched with non‐users on measures of disease progression and demographics.

**Results:**

In the primary analysis there were no significant differences in the trajectories between memantine users and non‐users on any primary outcomes of interest.

**Conclusions:**

Memantine use was not associated with any clinical benefit for individuals with manifest HD. Further studies are warranted to assess the impact of memantine on clinical outcomes in HD.

Memantine is an uncompetitive antagonist of the *N*‐methyl‐*d*‐aspartate (NMDA) receptor^1^ approved to treat moderate to severe dementia of the Alzheimer's type.^2^, ^3^ The NMDA receptor is a ligand‐gated ion channel that responds to glutamate, which is the primary excitatory neurotransmitter in the brain.^4^ Glutamate transmission plays an important role in learning, memory processing, and neural plasticity.^5^ Excessive glutamatergic neurotransmission is implicated in the pathology of Alzheimer's Disease (AD); however, normal physiologic levels of glutamate transmission are required for cognitive processing. Memantine is unique relative to other NMDA receptor‐active medications in that it acts as a noncompetitive antagonist with relatively low affinity for the NMDA receptor. This prevents prolonged receptor blockade that is associated with negative side effects on memory and cognition in higher affinity NMDA receptor antagonists, such as ketamine.^6^ Rather, memantine is thought to exert its positive effects in AD by blocking excessive glutamate transmission at the NMDA receptor while still allowing for normal glutamate levels.^1^

Huntington's Disease (HD) is a neurodegenerative disease characterized by progressively worsening motor symptoms, functionality, and cognition.^7^ Medium spiny neurons (MSNs) in the striatum are primarily impacted in patients with HD. MSNs are GABAergic and express NMDA receptors.^8^ It has been hypothesized that an abnormally high level of glutamate signaling may make MSNs more susceptible to neurodegeneration.^9^ Consequently, memantine has been hypothesized to treat the cognitive symptoms of HD or even slow neurodegeneration. A preclinical study using YAC128 transgenic mice showed that memantine was neuroprotective,^10^ but these results seemed to be dose‐dependent.^10^ These findings prompted human studies with conflicting results. One study followed 27 patients with HD for 2 years with doses up to 30 mg/day administered. The patients did not exhibit significant progression of any clinical outcome measures over time, but this study was not placebo‐controlled.^11^ Another study followed 12 patients with HD for 3 months after being titrated to 20 mg/day of memantine and no significant changes in cognitive or functional scores were noted.^12^ Records of two additional clinical trials are available, but efficacy results have not been posted (NCT00652457 & NCT01458470).

Given the uncertainty surrounding memantine in HD, we leveraged the Enroll‐HD database to investigate the cognitive effects of memantine on patients with HD. The primary aim of this study was to assess differences in clinical progression of cognitive symptoms in HD patients using memantine versus those not using memantine. These results have the potential to significantly impact clinical care that patients with HD receive using real‐world clinical data.

## Methods

Data from the 5th periodic dataset from the Enroll‐HD study were used for this analysis (https://enroll-hd.org).^13^ We identified participants with manifest HD defined as a CAG between 36 and 59 and a total motor score (TMS) greater than or equal to 10 or a total functional capacity (TFC) score less than 13 from the Unified Huntington's Disease Rating Scale at their index visit.^14^ Participants were excluded if they were using an acetylcholinesterase inhibitor within 180 days of their index visit or if they had epilepsy or a seizure disorder listed as a comorbidity. All participants were required to have at least one additional visit within 5 years of their index visit.

Memantine use was determined through identifying memantine as an ingredient in the pharmacologic dataset. Non‐users consisted of those who had never used memantine or any qualifying study visits prior to the initiation of memantine for those who used memantine. Those using memantine were grouped into prevalent users and incident users. A first‐treatment‐carried‐forward method was used for this study where any visits after initiation of the memantine medication were considered to be user visits. Further information on memantine users and non‐users can be found in the supplemental materials.

The primary outcomes for this study were cognitive measures, including the Symbol Digit Modalities Test (SDMT), Stroop Color Naming Test (SCNT), Stroop Word Reading Test (SWRT), and Stroop Interference Test (SIT). The TFC score was also included as a primary outcome due to the correlation between cognition and function.^15^ Secondary cognitive outcomes were also explored. Information can be found in the supplemental materials. The standardized mean difference (SMD) was used to compare users and non‐users at their index visit. SMD values less than 0.1 indicated balance between the groups. Memantine users were then matched to non‐users using nearest neighbor propensity score matching in a 2:1 ratio using the MatchIt package in R.^16^ For the primary analysis, participants were matched on age, sex, educational attainment, CAG repeat length, CAG‐Age‐Product (CAP)^17^ score, TMS, TFC score, benzodiazepine use, antipsychotic use, and number correct on the SDMT, SCNT, SWRT, and SIT.

Linear mixed effect models with a random intercept were used to assess differences in trajectories between memantine users and non‐users. Models included time, a time and memantine use interaction, and time on medication in addition to any variable that was not under the 0.1 SMD threshold after matching. Time was treated as a continuous variable rather than as set annual intervals to account for participants seen more or less frequently than on an annual basis. A secondary analysis compared incident users of memantine to non‐users as described above. This analysis allowed observation of clinical changes at the initiation of memantine. Two post‐hoc analyses were conducted to better understand the trajectories of the primary outcomes prior to initiating memantine. The details of these analyses can be found in the supplemental materials. All continuous variables in any of the models were centered around the mean of the participants in the model. All *P*‐values were false discovery rate (FDR)‐adjusted to account for multiple testing. An FDR‐adjusted *P*‐value of 0.05 indicated statistical significance. R version 4.0.5 was used to perform all analyses.

## Results

We identified 96 qualifying memantine users and 5745 non‐users of memantine (Table 1). There was no significant difference in the average number of follow‐up visits between groups (average = 3.3 visits, *P* = 0.218). For memantine users, the average amount of time using the medication prior to the index visit was 1.1 years. The percentage of memantine users still taking memantine at their last follow‐up visit was 64.6%.

**TABLE 1:** Participant Characteristics at Index Visit Pre‐ and Post‐Matching

	Pre‐Matching			Post‐Matching		
	Non‐Users *N* = 5745	Memantine Users *N* = 96	SMD	Non‐Users *N* = 192	Memantine Users *N* = 96	SMD
Mean (SD)						
Age, Years	51.1 (12.3)	52.5 (11.5)	0.121	53.7 (11.7)	52.5 (11.5)	0.101
CAG Repeat Length	43.6 (3.3)	43.8 (3.1)	0.044	43.7 (3.1)	43.8 (3.1)	0.037
CAP Score	479.6 (91.7)	502.6 (80.0)	0.268	509.3 (89.8)	502.6 (80.0)	0.078
SDMT Correct	27.1 (12.4)	25.1 (10.9)	0.172	23.9 (11.4)	25.1 (10.9)	0.105
SCNT Correct	47.8 (16.2)	44.0 (14.7)	0.248	43.0 (14.5)	44.0 (14.7)	0.068
SWRT Correct	63.5 (20.7)	60.3 (18.6)	0.161	59.3 (19.3)	60.3 (18.6)	0.055
SIT Correct	26.2 (11.7)	25.2 (10.4)	0.091	24.6 (17.4)	25.2 (10.4)	0.036
Total Motor Score	28.0 (15.7)	32.7 (16.9)	0.288	33.7 (18.1)	32.7 (16.9)	0.061
Total Functional Capacity Score	9.8 (2.7)	8.6 (2.7)	0.445	8.5 (3.1)	8.6 (2.7)	0.012
Time on Medication, Years	0.0 (0.0)	1.1 (1.2)	NA	0.0 (0.0)	1.1 (1.2)	NA
N (%)						
Educational Attainment			0.300			0.186
Less Than High School	1212 (21.1)	11 (11.5)		30 (15.6)	11 (11.5)	
High School Graduate	1940 (33.8)	32 (33.3)		60 (31.2)	32 (33.3)	
Associate's/Bachelor's Degree	2451 (42.7)	48 (50.0)		86 (44.4)	48 (50.0)	
Graduate Education	142 (2.5)	5 (5.2)		16 (8.3)	5 (5.2)	
Sex, Female	2943 (51.2)	45 (46.9)	0.087	84 (43.8)	45 (46.9)	0.063
Region			0.558			0.064
Europe	3848 (67.0)	46 (47.9)		94 (49.0)	46 (47.9)	
Northern America	1631 (28.4)	47 (49.0)		90 (46.9)	47 (49.0)	
Australasia	233 (4.1)	0 (0.0)		0 (0.0)	0 (0.0)	
Latin America	33 (0.6)	3 (3.1)		8 (4.2)	3 (3.1)	
Benzodiazepine Use	850 (14.8)	26 (27.1)	0.306	48 (25.0)	26 (27.1)	0.047
Antipsychotic Use	1527 (26.6)	45 (46.9)	0.431	86 (44.8)	45 (46.9)	0.042

Abbreviations: CAP, CAG‐Age‐Product; SCNT, Stroop Color Naming Test; SDMT, Symbol Digit Modality Test; SIT, Stroop Interference Test; SMD, Standardized Mean Difference; SWRT, Stroop Word Reading Test.

**Figure 1:**
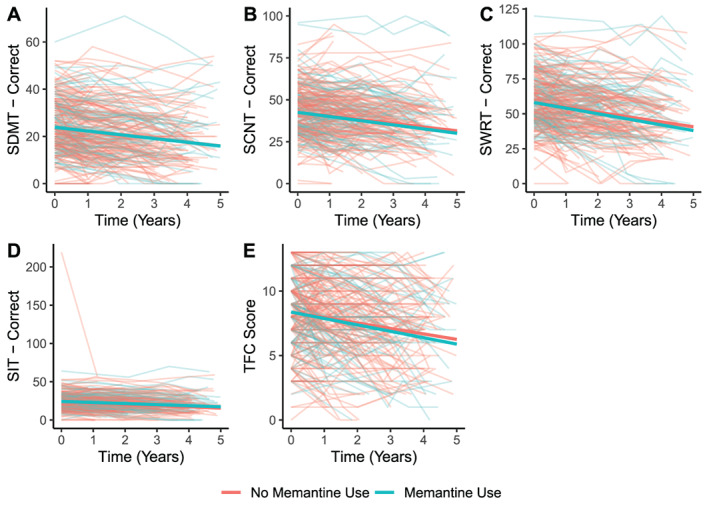
Differences in Trajectory of Clinical Outcome Measures Between Matched Memantine Users and Non‐Users. There were no differences in the trajectories between memantine users and non‐users for any of the primary outcomes. SCNT, stroop color naming test; SDMT, symbol digit modalities test; SIT, stroop interference test; SWRT, stroop word reading test; TFC, total functional capacity.

There were 96 memantine users matched to 192 non‐users (Table 1). The trajectories for memantine users and non‐users did not significantly differ for any of the primary outcome measures (Table S1, Fig. 1). For the secondary outcomes, the range of memantine users with complete data was between 55 and 84 users (Table S2). Memantine users had a significant decrease of 0.8 points greater per year than non‐users on the Letters Verbal Fluency Test, indicating enhanced worsening over that period of time (*P* = 0.013, Table S3, Fig. S1). The difference in trajectories was not significantly different between memantine users and non‐users for any other secondary outcome.

There were 17 incident users matched to 34 non‐users (Table S4). There were no significant differences in the trajectories of any primary outcome measures between incident users and non‐users (Table S5, Fig. S2). For the secondary outcomes, the number of incident users with complete data ranged from 9 to 14 (Table S6). There were no significant differences in trajectories of any secondary outcome measures between incident users and non‐users (Fig. S3, Table S7).

In the post‐hoc analyses, those who went on to use memantine had a significant decrease in the SWRT score of two points greater per year than non‐users, indicating a greater worsening of that measure (Table S8). There were no other significant differences in trajectories prior to medication initiation for any other primary outcomes. When only assessing the change in trajectory for memantine users from before use to after use, there were no significant changes in the trajectories for any primary outcome measures (Fig. S4, Table S9).

## Discussion

To the best of our knowledge, this is the largest reported analysis of the clinical effectiveness of memantine in patients with HD. We found that memantine use was not associated with any clinical benefit for individuals with HD. The two previous published studies of patients taking memantine^11^, ^12^ did not include a placebo group or a control group. While our study was retrospective, the use of propensity score matching allowed us to include a matched cohort of participants who were not being treated with memantine to make adequate comparisons between groups. Our results are in‐line with previous findings. A small placebo‐controlled study of memantine in HD was conducted to measure putaminal total *N*‐acetyl aspartate (tNAA) using magnetic resonance spectroscopy (NCT01458470). Eighteen early HD participants were enrolled, but only eight (four treated with memantine 20 mg/day and four treated with placebo for 6 months) participants completed the neuroimaging study. Available results from this study are limited, but the authors concluded that the results do not support the use of memantine in patients with early HD.^18^

This study does have limitations. First, we used a non‐user comparison group which may include individuals with different health‐seeking behaviors or less severe cognitive impairments. Matching users to non‐users was performed to account for these potential differences in disease symptomology. Additionally, a post‐hoc analysis assessing the differences in trajectories between non‐users and memantine was performed. Another limitation is the inclusion of prevalent users in the memantine user group. Prevalent users may include individuals who are more likely to tolerate treatment or those with greater health‐seeking behaviors. A secondary analysis with only incident users was performed to address this limitation. Another potential limitation is the inability to assess clinician preference and prescribing patterns for cognitive impairment in HD. It is unclear how many clinicians consider memantine as an option for HD patients with cognitive symptoms. However, based on the sample of 92 users in the large Enroll‐HD database, this may be an indication that memantine is not commonly considered for disease management. Additionally, medication data were self‐reported, which may introduce inaccuracies. Finally, the time between study visits is approximately 1 year in length. This long of a gap in visits may not adequately capture the effect of the medication.

In conclusion, this study suggests that memantine provides no clinical benefit for patients with HD. While clinical decision‐making should be individualized, these results support clinicians considering discontinuation of memantine in patients with HD. Further studies are warranted to assess the impact of memantine on clinical outcomes in HD.

## Author Roles

(1) Research project: A. Conception, B. Organization, C. Execution; (2) Statistical Analysis: A. Design, B. Execution, C. Review and Critique; (3) Manuscript Preparation: A. Writing of the first draft, B. Review and Critique.

A.C.O.: 1B, 1C, 2A, 2B, 3A, 3B

J.L.S.: 1A, 1B, 1C, 2A, 2C, 3A, 3B

## Disclosures

**Ethical Compliance Statement:** All Enroll‐HD sites are required to obtain and maintain local ethical approval. Use of data from this study was reviewed by our Institutional Review Board and determined to be secondary data analysis of de‐identified data and not human subjects research. As a result, IRB approval was not required, but the Chair of the IRB Committee provided confirmation that it was ethical for us to conduct this retrospective research.

**Funding Sources and Conflicts of Interest:** No specific funding was received for this work. The authors declare that there are no conflicts of interest relevant to this work.

**Financial Disclosures for the Previous 12 Months:** The authors declare that there are no additional disclosures to report.

## Supporting information


**Data S1.** Supplemental Materials—Methods and Discussion

**Table S1.** Prevalent User Matched Model Output. Table S1 presents the model estimates and false discovery rate values for each primary outcome of interest when including prevalent users and matched non‐users
**Table S2.** Pre‐ and Post‐Matching Sample Size and Secondary Outcome Values for Memantine Users and Non‐Users. Supplemental Table S2 presents the sample sizes for each secondary outcome before and after matching. Additionally, the table presents the mean scores, standard deviations, and standardized mean differences for each secondary outcome before and after matching
**Table S3.** Secondary Outcomes Model Output for Memantine Users and Non‐Users. Supplemental Table S3 presents the model results for the intercept, time, and interaction between time and memantine use for each secondary outcome. As the unbalanced variables for each secondary outcome differed, these variables are listed in the table notes
**Table S4.** Participant Characteristics at Index Visit Pre‐ and Post‐Matching for Incident Users and Non‐Users. Supplemental Table S4 presents the demographic and clinical characteristics for incident memantine users and non‐users both pre‐ and post‐matching
**Table S5.** Incident User Matched Model Output. Supplemental Table S5 presents the model estimates and false discovery rate values for each primary outcome of interest when including incident users and matched non‐users
**Table S6.** Pre‐ and Post‐Matching Sample Size and Secondary Outcome Values for Incident Memantine Users and Non‐Users. Supplemental Table S6 presents the sample sizes for each secondary outcome before and after matching. Additionally, the table presents the mean scores, standard deviations, and standardized mean differences for each secondary outcome before and after matching
**Table S7.** Secondary Outcomes Model Output for Incident Memantine Users and Non‐Users. Supplemental Table S7 presents the model results for the intercept, time, and interaction between time and memantine use for each secondary outcome. As the unbalanced variables for each secondary outcome differed, these variables are listed in the table notes
**Table S8.** Model Output for Comparing Pre‐Memantine Use Trajectories of Primary Outcomes to Non‐User Trajectories. Supplemental Table S8 presents the model estimates and false discover rate values for each primary outcome for memantine users prior to initiating memantine and non‐users
**Table S9.** Model Output for Comparing Trajectories of Primary Outcomes Before and After Initiating Memantine for Memantine Users. Supplemental Table S9 presents the model estimates and false discovery rate values for each primary outcome for comparing the trajectories of decline prior to initiating memantine and after memantine initiation. A significant difference in trajectory after initiating memantine would be indicated by a significant *P*‐value for the spline term of the model
**Figure S1.** Differences in Trajectory of Secondary Outcome Measures Between Matched Memantine and Non‐Memantine Users. Memantine users had a significantly greater rate of decline by 0.8 points of the VERFLT compared to non‐users. There were no other significant differences in the trajectories for any other secondary outcome. VERFLT, Letters Verbal Fluency Test; VERFCT, Categorical Verbal Fluency Test; TRLA, Trail Making Test Part A; TRLB, Trail Making Test Part B; MMSE, Mini Mental State Examination
**Figure S2.** Differences in Trajectory of Primary Outcome Measures Between Matched Incident Memantine and Non‐Memantine Users. There were no significant differences in the trajectories of any primary outcome measures between incident users and non‐users. VERFLT, Letters Verbal Fluency Test; VERFCT, Categorical Verbal Fluency Test; TRLA, Trail Making Test Part A; TRLB, Trail Making Test Part B; MMSE, Mini Mental State Examination
**Figure S3.** Differences in Trajectory of Secondary Outcome Measures Between Matched Incident Memantine and Non‐Memantine Users. There were no significant differences in the trajectories of any secondary outcome measures between incident users and non‐users. VERFLT, Letters Verbal Fluency Test; VERFCT, Categorical Verbal Fluency Test; TRLA: Trail Making Test Part A; TRLB: Trail Making Test Part B; MMSE: Mini Mental State Examination
**Figure S4.** Differences in Trajectory of Clinical Outcome Measures Before and After Memantine Initiation in Memantine Users. There were no differences in trajectories before and after memantine initiation for any of the primary outcomes. SCNT: Stroop Color Naming Test; SDMT: Symbol Digit Modalities Test; SIT: Stroop Interference Test; SWRT, Stroop Word Reading Test; TFC, Total Functional Capacity
