# Supplementary material for: Memantine Use and Cognitive Decline in Huntington's Disease: An Enroll‐HD Study
Source: Mov Disord Clin Pract. 2023 May 16;10(7):1120–5. doi: 10.1002/mdc3.13763 (PMC10354618; doi:10.1002/mdc3.13763)
Supplement: Supplementary file 1 — Data S1. Supplemental Materials—Methods and Discussion [file MDC3-10-1120-s002.docx]

**Memantine Use Determination**

A participant’s earliest memantine medication use period was used for this study. Participants were excluded from the study if they had a missing start day, missing continued use indicator, or missing end day if the medication had been discontinued. The index visit for non-users was the first manifest HD visit where complete data were available for the variables of interest listed previously. The index visit for prevalent users was the first manifest HD visit with complete data after initiating the medication. The index visit for incident users was required to be no more than 90 days prior to the initiation of the medication.

**Secondary Analysis Outcome Variables and Analysis**

Secondary analysis outcome variables included the Letters Verbal Fluency Test, the Categorical Verbal Fluency Test, Parts A and B of the Trail Making Test, and the Mini Mental State Examination score. For the secondary outcomes, the matching process was completed for each measure individually to maximize the number of participants without missing data.

**Post-Hoc Analyses**

The first post-hoc analysis used linear mixed effect models to compare the trajectories of the primary cognitive measures in memantine users prior to initiating the medication to the trajectories of these measures in non-users. The second post-hoc analysis used data only from memantine users to look at the trajectories of the primary outcome measures both before and after initiation of the medication. In this case, a piecewise linear mixed effect model was used with a knot at the time of medication initiation.

**Latrepirdine and HD**

Two randomized controlled trials have been conducted to test the effect of latrepirdine on cognition and global function in patients with HD.^19, 20^ This is important because latrepirdine has been shown to act as an inhibitor of NMDA receptors and voltage-gated calcium channels.^21^ Latrepirdine was well-tolerated in the initial Phase 2 study and seemed to be associated with some improvement in cognitive ability.^19^ Unfortunately, this medication failed to show significant benefits on cognition and global ability in a follow-up Phase 3 study in patients with HD.^20^

**Comparison to Studies in Alzheimer’s Disease**

There are some key differences between our current analysis and the prospective studies of memantine in both AD and HD. The approval of memantine for the treatment of AD was based on studies that were 24 to 28 weeks long.^1, 2^ The longest follow-up period for studies examining memantine in patients with HD was two years.^3^ In contrast, our current study follows participants for up to five years since entry into the Enroll-HD study. Additionally, memantine has been shown to be more effective in treating AD relative to placebo when added to an established regimen of an acetylcholinesterase inhibitor.^2^ Two previous studies have failed to show any significant improvement in cognitive symptoms of HD in patients treated with the acetylcholinesterase inhibitors donepezil^4^ or rivastigmine.^5^ Our study only evaluated participants known to be using memantine independent of their use of an acetylcholinesterase inhibitor. The combination of these treatments may be associated with improved cognitive outcomes, as has been demonstrated in patients with AD.

References

1. Reisberg B, Doody R, Stöffler A, Schmitt F, Ferris S, Möbius HJ. Memantine in moderate-to-severe Alzheimer's disease. N Engl J Med 2003;348:1333-1341.

2. McShane R, Westby MJ, Roberts E, et al. Memantine for dementia. Cochrane Database Syst Rev 2019;3:Cd003154.

3. Beister A, Kraus P, Kuhn W, Dose M, Weindl A, Gerlach M. The N-methyl-D-aspartate antagonist memantine retards progression of Huntington's disease. J Neural Transm Suppl 2004:117-122.

4. Cubo E, Shannon KM, Tracy D, et al. Effect of donepezil on motor and cognitive function in Huntington disease. Neurology 2006;67:1268-1271.

5. Sešok S, Bolle N, Kobal J, Bucik V, Vodušek DB. Cognitive function in early clinical phase huntington disease after rivastigmine treatment. Psychiatr Danub 2014;26:239-248.
