# Supplementary material for: Memantine Use and Cognitive Decline in Huntington's Disease: An Enroll‐HD Study
Source: Mov Disord Clin Pract. 2023 May 16;10(7):1120–5. doi: 10.1002/mdc3.13763 (PMC10354618; doi:10.1002/mdc3.13763)
Supplement: Supplementary file 2 — Table S1. Prevalent User Matched Model Output. Table S1 presents the model estimates and false discovery rate values for each primary outcome of interest when including prevalent users and matched non‐users Table S2. Pre‐ and Post‐Matching Sample Size and Secondary Outcome Values for Memantine Users and Non‐Users. Supplemental Table S2 presents the sample sizes for each secondary outcome before and after matching. Additionally, the table presents the mean scores, standard deviations, and standardized mean differences for each secondary outcome before and after matching Table S3. Secondary Outcomes Model Output for Memantine Users and Non‐Users. Supplemental Table S3 presents the model results for the intercept, time, and interaction between time and memantine use for each secondary outcome. As the unbalanced variables for each secondary outcome differed, these variables are listed in the table notes Table S4. Participant Characteristics at Index Visit Pre‐ and Post‐Matching for Incident Users and Non‐Users. Supplemental Table S4 presents the demographic and clinical characteristics for incident memantine users and non‐users both pre‐ and post‐matching Table S5. Incident User Matched Model Output. Supplemental Table S5 presents the model estimates and false discovery rate values for each primary outcome of interest when including incident users and matched non‐users Table S6. Pre‐ and Post‐Matching Sample Size and Secondary Outcome Values for Incident Memantine Users and Non‐Users. Supplemental Table S6 presents the sample sizes for each secondary outcome before and after matching. Additionally, the table presents the mean scores, standard deviations, and standardized mean differences for each secondary outcome before and after matching Table S7. Secondary Outcomes Model Output for Incident Memantine Users and Non‐Users. Supplemental Table S7 presents the model results for the intercept, time, and interaction between time and memantine use for each secondary outcome. [file MDC3-10-1120-s001.docx]

**Supplemental Table S1 Model Estimates for Matched Memantine Users and Non-Users**

|  | **SDMT Model** | | **SCNT Model** | | **SWRT Model** | | **SIT Model** | | **TFC Model** | |
| --- | --- | --- | --- | --- | --- | --- | --- | --- | --- | --- |
|  | Estimate | P Value | Estimate | P Value | Estimate | P Value | Estimate | P Value | Estimate | P Value |
| Intercept | 23.9 | **< 0.001** | 42.5 | **< 0.001** | 58.1 | **< 0.001** | 24.3 | **< 0.001** | 8.4 | **< 0.001** |
| Time (Years) | -1.6 | **< 0.001** | -2.2 | **< 0.001** | -3.5 | **< 0.001** | -1.8 | **< 0.001** | -0.4 | **< 0.001** |
| Time on Medication (Years) | 0.2 | 0.686 | 0.5 | 0.686 | 0.3 | 0.921 | -0.1 | 0.935 | -0.3 | 0.109 |
| Education – Less Than High School | 1.0 | 0.337 | 5.7 | **0.006** | 5.1 | 0.105 | 2.8 | 0.105 | 0.8 | 0.153 |
| Education – High School Graduate | 0.1 | 0.935 | 0.6 | 0.863 | 0.7 | 0.863 | 0.4 | 0.863 | 0.5 | 0.310 |
| Education – Graduate Education | -0.7 | 0.674 | -2.3 | 0.556 | 0.1 | 0.974 | -0.3 | 0.940 | -0.1 | 0.935 |
| Age | 0.0 | 0.863 | -0.0 | 0.824 | 0.0 | 0.957 | -0.2 | **< 0.001** | -0.0 | 0.921 |
| SDMT Score | 0.9 | **< 0.001** | 1.0 | **< 0.001** | 1.3 | **< 0.001** | 0.7 | **< 0.001** | 0.2 | **< 0.001** |
| Time * Memantine Use | -0.0 | 0.971 | -0.3 | 0.621 | -0.5 | 0.469 | 0.4 | 0.540 | -0.1 | 0.569 |

SDMT – Symbol Digit Modalities Test; SCNT – Stroop Color Naming Test; SWRT – Stroop Word Reading Test;

SIT – Stroop Interference Test; TFC – Total Functional Capacity

**Supplemental Table S2**

|  | Pre-Match Non-Users (N) | Pre-Match Memantine Users (N) | Pre-Match Non-User Score (Mean (SD)) | Pre-Match Memantine User Score (Mean (SD)) | Pre-Match SMD | Post-Match Non-Users (N) | Post-Match Memantine Users (N) | Post-Match Non-User Score (Mean (SD)) | Post-Match Memantine User Score (Mean (SD)) | Post-Match SMD |
| --- | --- | --- | --- | --- | --- | --- | --- | --- | --- | --- |
| Verbal Fluency Test Letters Correct | 4998 | 75 | 23.5 (12.5) | 22.9 (13.2) | 0.046 | 144 | 74 | 22.0 (11.9) | 23.0 (13.2) | 0.073 |
| Verbal Fluency Test Category Correct | 4949 | 84 | 14.0 (5.6) | 13.1 (5.2) | 0.169 | 163 | 83 | 12.9 (5.4) | 13.0 (5.2) | 0.018 |
| Time to Complete Trail Making Part A | 3780 | 62 | 60.7 (40.7) | 63.3 (37.8) | 0.065 | 115 | 58 | 66.2 (46.8) | 63.6 (38.9) | 0.060 |
| Time to Complete Trail Making Part B | 3691 | 59 | 137.8 (69.5) | 132.8 (67.4) | 0.074 | 112 | 57 | 125.5 (65.0) | 134.3 (68.1) | 0.132 |
| Mini Mental State Examination | 2751 | 55 | 26.2 (3.2) | 26.2 (3.3) | 0.004 | 101 | 51 | 26.3 (3.3) | 26.1 (3.4) | 0.032 |

SMD – Standardized Mean Difference

**Supplemental Table S3**

|  | **VERFLT Model^a^** | | **VERFCT Model^b^** | | | **Trails A Model^c^** | | | **Trails B Model^d^** | | | **MMSE Model^e^** | | |  |
| --- | --- | --- | --- | --- | --- | --- | --- | --- | --- | --- | --- | --- | --- | --- | --- |
|  | Estimate | P Value | | Estimate | P Value | | Estimate | P Value | | Estimate | P Value | | Estimate | P Value | |
| Intercept | 21.8 | **< 0.001** | | 14.9 | **< 0.001** | | 62.2 | **< 0.001** | | 141.0 | **< 0.001** | | 26.8 | **< 0.001** | |
| Time (Years) | -0.6 | **< 0.001** | | -0.7 | **< 0.001** | | 4.4 | **< 0.001** | | 7.3 | **< 0.001** | | -0.4 | **< 0.001** | |
| Time*Memantine Use | -0.8 | **0.013** | | 0.1 | 0.571 | | -3.3 | 0.063 | | 3.2 | 0.206 | | 0.1 | 0.571 | |

^a^ Model adjusted for CAG repeat length, CAP score, region, benzodiazepine use, and antipsychotic use

^b^ Model adjusted for age, CAG repeat length, educational attainment, region, and antipsychotic use

^c^ Model adjusted for age, CAG repeat length, educational attainment, and region

^d^ Model adjusted for educational attainment, region, SIT score, time to complete TRLB, and benzodiazepine use

^e^ Model adjusted for educational attainment, SIT score, and antipsychotic use

VERFLT: Letters Verbal Fluency Test

VERFCT: Categorical Verbal Fluency Test

MMSE: Mini Mental State Examination

CAP: CAG-Age-Product

TRLB: Trail Making Part B

**Supplemental Table S4 Participant Characteristics at Index Visit Pre- and Post-Matching for Incident Users and Non-Users**

|  | **Pre-Matching** | | |  | **Post-Matching** | | |
| --- | --- | --- | --- | --- | --- | --- | --- |
|  | Non-Users | Memantine Users | SMD |  | Non-Users | Memantine Users | SMD |
|  | N = 5745 | N = 17 |  |  | N = 34 | N = 17 |  |
| **Mean (SD)** |  |  |  |  |  |  |  |
| Age, Years | 51.1 (12.3) | 49.7 (13.6) | 0.105 |  | 46.4 (15.4) | 49.7 (13.6) | 0.231 |
| CAG Repeat Length | 43.6 (3.3) | 44.4 (4.3) | 0.201 |  | 44.9 (4.5) | 44.4 (4.3) | 0.100 |
| CAP Score | 479.6 (91.7) | 486.0 (86.8) | 0.072 |  | 463.4 (86.2) | 486.0 (86.8) | 0.262 |
| SDMT Correct | 27.1 (12.4) | 26.3 (9.0) | 0.073 |  | 29.1 (12.6) | 26.3 (9.0) | 0.256 |
| SCNT Correct | 47.8 (16.2) | 48.1 (12.0) | 0.017 |  | 49.5 (14.4) | 48.1 (12.0) | 0.109 |
| SWRT Correct | 63.5 (20.7) | 62.3 (16.7) | 0.062 |  | 62.4 (19.8) | 62.3 (16.7) | 0.006 |
| SIT Correct | 26.2 (11.7) | 28.2 (10.1) | 0.184 |  | 29.2 (9.4) | 28.2 (10.1) | 0.100 |
| Total Motor Score | 28.0 (15.7) | 23.0 (11.9) | 0.356 |  | 19.9 (10.5) | 23.0 (11.9) | 0.275 |
| Total Functional Capacity Score | 9.8 (2.7) | 9.6 (2.5) | 0.078 |  | 10.2 (2.5) | 9.6 (2.5) | 0.258 |
| **N (%)** |  |  |  |  |  |  |  |
| Educational Attainment |  |  | 0.788 |  |  |  | 0.179 |
| Less Than High School | 1212 (21.1) | 0 (0.0) |  |  | 0 (0.0) | 0 (0.0) |  |
| High School Graduate | 1940 (33.8) | 8 (47.1) |  |  | 13 (38.2) | 8 (47.1) |  |
| Associate’s/Bachelor’s Degree | 2451 (42.7) | 9 (52.9) |  |  | 21 (61.8) | 9 (52.9) |  |
| Graduate Education | 142 (2.5) | 0 (0.0) |  |  | 0 (0.0) | 0 (0.0) |  |
| Sex, Female | 2943 (51.2) | 6 (35.3) | 0.326 |  | 9 (26.5) | 6 (35.3) | 0.192 |
| Region |  |  | 0.395 |  |  |  | 0.315 |
| Europe | 3848 (67.0) | 10 (58.8) |  |  | 25 (73.5) | 10 (58.8) |  |
| Northern America | 1631 (28.4) | 7 (41.2) |  |  | 9 (26.5) | 7 (41.2) |  |
| Australasia | 233 (4.1) | 0 (0.0) |  |  | 0 (0.0) | 0 (0.0) |  |
| Latin America | 33 (0.6) | 0 (0.0) |  |  | 0 (0.0) | 0 (0.0) |  |
| Benzodiazepine Use | 850 (14.8) | 1 (5.9) | 0.296 |  | 1 (2.9) | 1 (5.9) | 0.144 |
| Antipsychotic Use | 1527 (26.6) | 7 (41.2) | 0.312 |  | 11 (32.4) | 7 (41.2) | 0.184 |

SMD – Standardized Mean Difference; CAP – CAG-Age-Product; SDMT – Symbol Digit Modality Test; SCNT – Stroop Color Naming Test; SWRT – Stroop Word Reading Test; SIT – Stroop Interference Test;

**Supplemental Table S5**

|  | **SDMT Model** | | | **SCNT Model** | | | **SWRT Model** | | | **SIT Model** | | | **TFC Model** | | |
| --- | --- | --- | --- | --- | --- | --- | --- | --- | --- | --- | --- | --- | --- | --- | --- |
|  | Estimate | P Value | Estimate | | P Value | Estimate | | P Value | Estimate | | P Value | Estimate | | P Value |  |
| Intercept | 30.1 | **< 0.001** | 49.7 | | **< 0.001** | 63.7 | | **< 0.001** | 29.9 | | **< 0.001** | 10.4 | | **< 0.001** |  |
| Time (Years) | -1.6 | **< 0.001** | -1.8 | | **< 0.001** | -2.3 | | **0.009** | -1.6 | | **< 0.001** | -0.8 | | **< 0.001** |  |
| Age | 0.1 | 0.679 | -0.1 | | 0.866 | 0.8 | | **0.048** | -0.1 | | 0.799 | -0.0 | | 0.989 |  |
| CAG Repeat Length | 0.5 | 0.572 | -0.5 | | 0.740 | 1.9 | | 0.228 | -0.1 | | 0.885 | 0.0 | | 0.989 |  |
| CAP Score | -0.0 | 0.426 | 0.0 | | 0.866 | -0.0 | | 0.574 | -0.0 | | 0.866 | -0.0 | | 0.866 |  |
| High School Graduate | -0.4 | 0.866 | 0.8 | | 0.866 | 1.2 | | 0.866 | 0.5 | | 0.866 | 0.1 | | 0.936 |  |
| Sex – Female | -2.1 | 0.373 | 1.4 | | 0.799 | 5.1 | | 0.345 | -0.1 | | 0.989 | -0.5 | | 0.574 |  |
| Region – Northern America | -1.4 | 0.686 | -1.8 | | 0.737 | -6.6 | | 0.214 | -1.6 | | 0.586 | 0.0 | | 0.989 |  |
| SDMT | 0.7 | **< 0.001** | 0.3 | | 0.214 | 0.3 | | 0.490 | 0.0 | | 0.866 | 0.0 | | 0.574 |  |
| SCNT | 0.0 | 0.996 | 0.6 | | **< 0.001** | 0.1 | | 0.714 | 0.1 | | 0.490 | -0.0 | | 0.866 |  |
| SIT | 0.1 | 0.771 | 0.1 | | 0.807 | 0.8 | | **0.012** | 0.7 | | **< 0.001** | -0.0 | | 0.989 |  |
| Total Motor Score | -0.2 | 0.214 | -0.1 | | 0.825 | -0.4 | | 0.102 | -0.0 | | 0.866 | -0.0 | | 0.737 |  |
| Total Functional Capacity Score | -0.5 | 0.282 | -0.2 | | 0.866 | -0.7 | | 0.574 | -0.3 | | 0.574 | 0.7 | | **< 0.001** |  |
| Benzodiazepine Use | 0.9 | 0.866 | 2.5 | | 0.825 | 5.2 | | 0.737 | 0.2 | | 0.989 | -0.6 | | 0.799 |  |
| Antipsychotic Use | -2.8 | 0.221 | -3.2 | | 0.470 | -2.9 | | 0.696 | -1.6 | | 0.574 | -0.5 | | 0.679 |  |
| Time*Memantine Use | 0.0 | 0.989 | -0.9 | | 0.542 | -0.9 | | 0.732 | 0.3 | | 0.807 | 0.2 | | 0.490 |  |

SDMT: Symbol Digit Modalities Test

SCNT: Stroop Color Naming Test

SWRT: Stroop Word Reading Test

SIT: Stroop Interference Test

TFC: Total Functional Capacity

CAP: CAG-Age-Product

**Supplemental Table S6**

|  | Pre-Match Non-Users (N) | Pre-Match Memantine Users (N) | Pre-Match Non-User Score (Mean (SD)) | Pre-Match Memantine User Score (Mean (SD)) | Pre-Match SMD | Post-Match Non-Users (N) | Post-Match Memantine Users (N) | Post-Match Non-User Score (Mean (SD)) | Post-Match Memantine User Score (Mean (SD)) | Post-Match SMD |
| --- | --- | --- | --- | --- | --- | --- | --- | --- | --- | --- |
| Verbal Fluency Test Letters Correct | 4998 | 14 | 23.5 (12.5) | 24.6 (13.2) | 0.083 | 28 | 14 | 22.6 (7.6) | 24.6 (13.2) | 0.186 |
| Verbal Fluency Test Category Correct | 4949 | 13 | 14.0 (5.6) | 13.5 (4.1) | 0.105 | 26 | 13 | 13.1 (4.1) | 13.5 (4.1) | 0.095 |
| Time to Complete Trail Making Part A | 3780 | 10 | 60.7 (40.7) | 44.0 (15.6) | 0.543 | 16 | 8 | 43.9 (13.7) | 47.3 (15.7) | 0.229 |
| Time to Complete Trail Making Part B | 3691 | 9 | 137.8 (69.5) | 123.6 (79.8) | 0.191 | 17 | 9 | 138.2 (71.5) | 123.6 (79.8) | 0.194 |
| Mini Mental State Examination | 2751 | 9 | 26.2 (3.2) | 27.2 (3.1) | 0.321 | 16 | 8 | 26.7 (2.1) | 27.0 (3.2) | 0.116 |

SMD – Standardized Mean Difference

**Supplemental Table S7**

|  | **VERFLT Model^a^** | | **VERFCT Model^b^** | | | **Trails A Model^c^** | | | **Trails B Model^d^** | | | **MMSE Model^e^** | | |  |
| --- | --- | --- | --- | --- | --- | --- | --- | --- | --- | --- | --- | --- | --- | --- | --- |
|  | Estimate | P Value | | Estimate | P Value | | Estimate | P Value | | Estimate | P Value | | Estimate | P Value | |
| Intercept | 24.7 | **< 0.001** | | 13.0 | **< 0.001** | | 34.1 | **< 0.001** | | 122.4 | **0.035** | | 24.6 | **< 0.001** | |
| Time (Years) | -0.2 | 0.910 | | -0.7 | **0.027** | | 7.6 | **0.001** | | 8.2 | 0.514 | | 0.0 | 0.955 | |
| Time*Memantine Use | -0.3 | 0.914 | | -0.4 | 0.629 | | -0.8 | 0.955 | | 19.5 | 0.335 | | -0.6 | 0.382 | |

^a^ Model adjusted for CAG repeat length, CAP score, TFC score, SDMT score, SCNT score, SWRT score, SIT score, VERFLT score, sex, benzodiazepine use, and antipsychotic use

^b^ Model adjusted for CAP score, educational attainment, SDMT score, SCNT score, SWRT score, SIT score, total motor score, TFC score, and benzodiazepine use

^c^ Model adjusted for age, CAG repeat length, CAP score, total motor score, TFC score, SWRT score, SIT score, time to complete TRLA, sex, region, educational attainment, benzodiazepine use, and antipsychotic use

^d^ Model adjusted for age, CAG repeat length, educational attainment, region, SCNT score, time to complete TRLB, total motor score, TFC score, benzodiazepine use, and antipsychotic use

^e^ Model adjusted for age, CAG repeat length, CAP score, educational attainment, sex, region, SCNT score, SWRT score, MMSE score, total motor score, TFC score, benzodiazepine use, and antipsychotic use

CAP: CAG-Age-Product

VERFLT: Letters Verbal Fluency Test

VERFCT: Categorical Verbal Fluency Test

MMSE: Mini Mental State Examination

TRLA: Trail Making Part A

TRLB: Trail Making Part B

**Supplemental Table S8**

|  | **SDMT Model** | | | **SCNT Model** | | | **SWRT Model** | | | **SIT Model** | | | **TFC Model** | | |
| --- | --- | --- | --- | --- | --- | --- | --- | --- | --- | --- | --- | --- | --- | --- | --- |
|  | Estimate | P Value | Estimate | | P Value | Estimate | | P Value | Estimate | | P Value | Estimate | | P Value |  |
| Intercept | 30.1 | **< 0.001** | 48.4 | | **< 0.001** | 65.9 | | **< 0.001** | 27.2 | | **< 0.001** | 10.6 | | **< 0.001** |  |
| Time (Years) | -1.6 | **< 0.001** | -1.9 | | **< 0.001** | -2.4 | | **< 0.001** | -0.7 | | **0.018** | -0.4 | | **< 0.001** |  |
| CAP Score | -0.0 | 0.055 | -0.0 | | 0.457 | 0.0 | | 0.966 | -0.0 | | 0.613 | 0.0 | | 0.750 |  |
| SDMT Score | 0.9 | **< 0.001** | 0.5 | | **< 0.001** | 0.2 | | **0.028** | 0.5 | | **< 0.001** | 0.0 | | 0.112 |  |
| SWRT Score | 0.0 | 0.191 | 0.4 | | **< 0.001** | 0.8 | | **< 0.001** | 0.1 | | 0.055 | 0.1 | | **0.001** |  |
| Education –  Less Than High School | 0.5 | 0.813 | 6.1 | | 0.055 | 0.1 | | 0.982 | 3.9 | | 0.217 | -0.2 | | 0.875 |  |
| Education –  High School Graduate | 1.1 | 0.259 | 1.0 | | 0.750 | 0.0 | | 0.982 | -0.6 | | 0.807 | 0.1 | | 0.823 |  |
| Education –  Graduate Education | 0.8 | 0.750 | 2.6 | | 0.647 | 2.1 | | 0.719 | 1.9 | | 0.723 | 0.9 | | 0.457 |  |
| Time*Memantine Use | -0.1 | 0.875 | 0.2 | | 0.823 | -2.0 | | **0.025** | -0.9 | | 0.229 | -0.1 | | 0.750 |  |

SDMT: Symbol Digit Modalities Test; SCNT: Stroop Color Naming Test

SWRT: Stroop Word Reading Test; SIT: Stroop Interference Test

TFC: Total Functional Capacity; CAP: CAG-Age-Product

**Supplemental Table S9**

|  | **SDMT Model** | | **SCNT Model** | | | **SWRT Model** | | | **SIT Model** | | | **TFC Model** | | |  |
| --- | --- | --- | --- | --- | --- | --- | --- | --- | --- | --- | --- | --- | --- | --- | --- |
|  | Estimate | P Value | | Estimate | P Value | | Estimate | P Value | | Estimate | P Value | | Estimate | P Value | |
| Intercept | 29.5 | 0.091 | | 43.8 | **0.041** | | 53.4 | **0.032** | | 25.5 | 0.064 | | 9.2 | 0.056 | |
| Time (Years) | -3.1 | **0.032** | | -2.9 | 0.086 | | -5.0 | 0.032 | | -1.3 | 0.541 | | -0.8 | **0.032** | |
| Time – Spline (Years) | 1.4 | 1.000 | | -1.1 | 1.000 | | 2.0 | 1.000 | | -1.0 | 1.000 | | 0.1 | 1.000 | |
| Age at Initiation | -0.7 | 1.000 | | -0.4 | 1.000 | | -0.5 | 1.000 | | -0.2 | 1.000 | | 0.1 | 1.000 | |
| CAG Repeat Length | -4.1 | 1.000 | | -3.3 | 1.000 | | -5.9 | **0.959** | | -1.5 | 1.000 | | -0.4 | 1.000 | |
| Sex – Male | -3.8 | 1.000 | | -5.3 | 1.000 | | -0.1 | 1.000 | | -0.2 | 1.000 | | -1.3 | 1.000 | |
| Education –  Less Than High School | -9.1 | 1.000 | | -0.5 | 1.000 | | -5.2 | 1.000 | | 0.7 | 1.000 | | -0.0 | 1.000 | |
| Education –  High School Graduate | 8.2 | 1.000 | | -0.0 | 1.000 | | 9.4 | 1.000 | | 1.8 | 1.000 | | 1.3 | 1.000 | |
| Education –  Graduate Education | -10.8 | 1.000 | | -3.6 | 1.000 | | 2.0 | 1.000 | | -6.5 | 1.000 | | 0.8 | 1.000 | |
| Region –  Northern America | -2.8 | 1.000 | | 4.7 | 1.000 | | 0.3 | 1.000 | | 1.6 | 1.000 | | 1.5 | 1.000 | |
| Age at Initiation*CAG Repeat Length | 0.1 | 1.000 | | -0.1 | 1.000 | | 0.1 | 1.000 | | 0.1 | 1.000 | | 0.0 | 1.000 | |

SDMT: Symbol Digit Modalities Test

SCNT: Stroop Color Naming Test

SWRT: Stroop Word Reading Test

SIT: Stroop Interference Test

TFC: Total Functional Capacity

**Supplemental Figure S1**

**
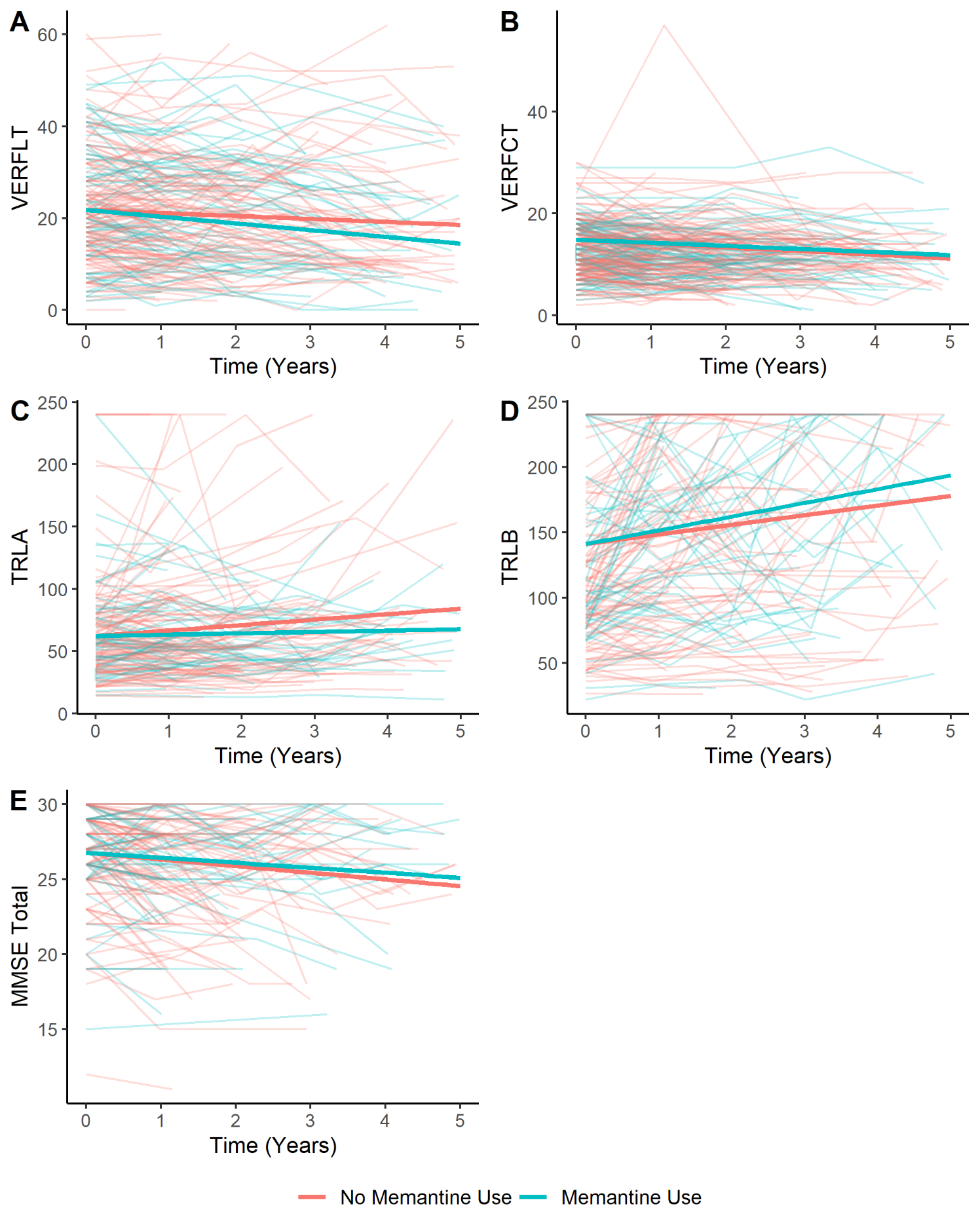
**

**Supplemental Figure S2**

**
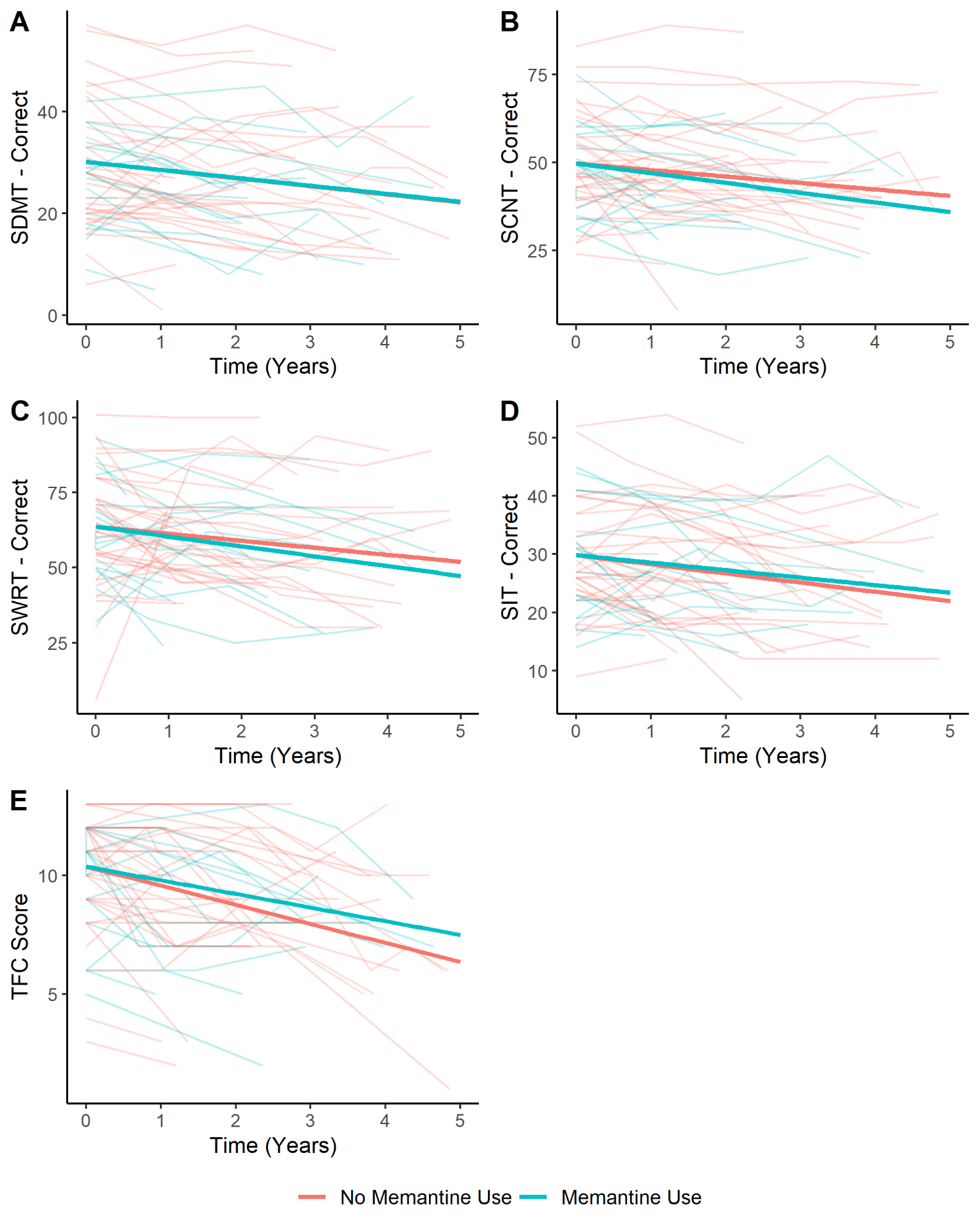
**

**Supplemental Figure S3**


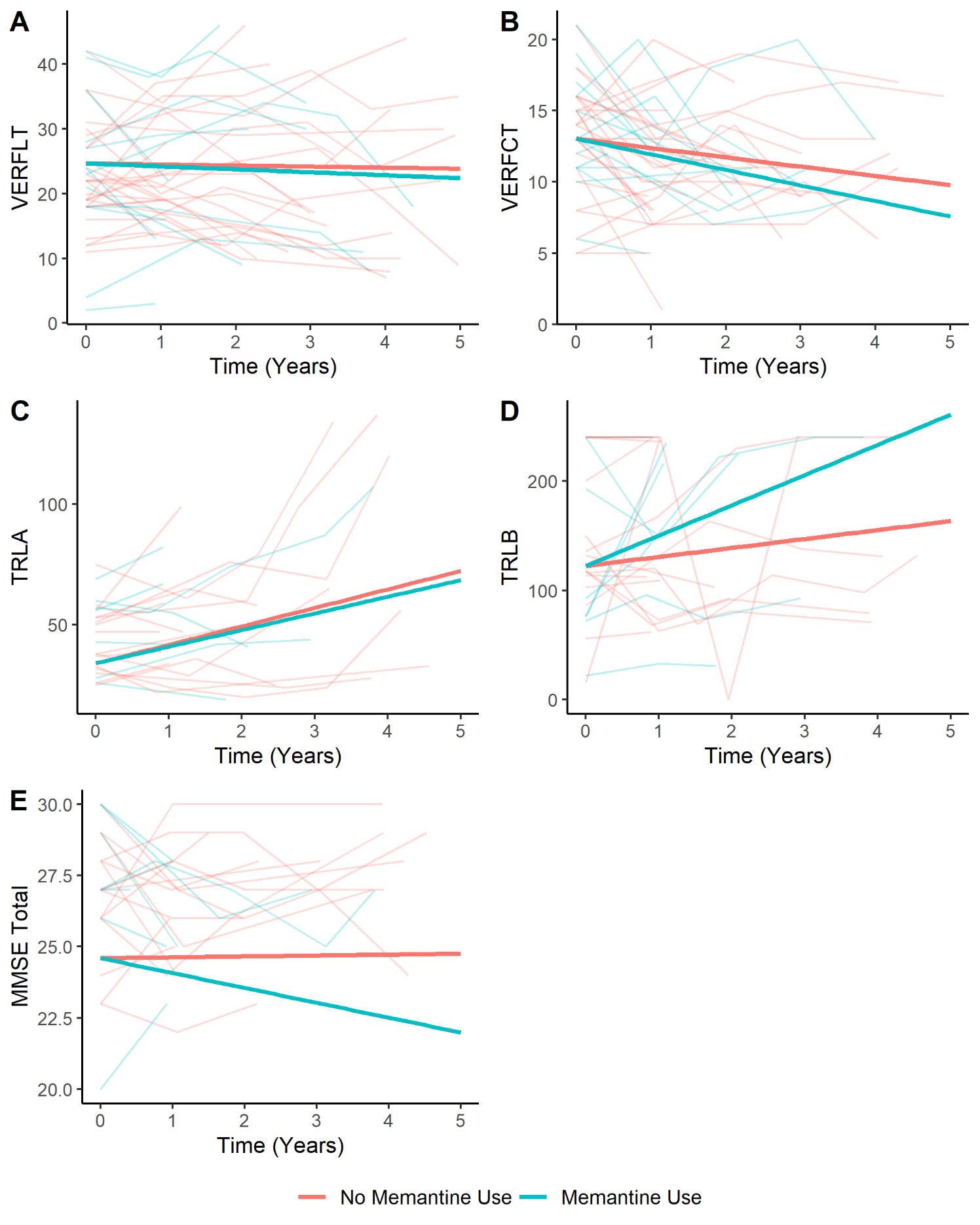


**Supplemental Figure S4**

**
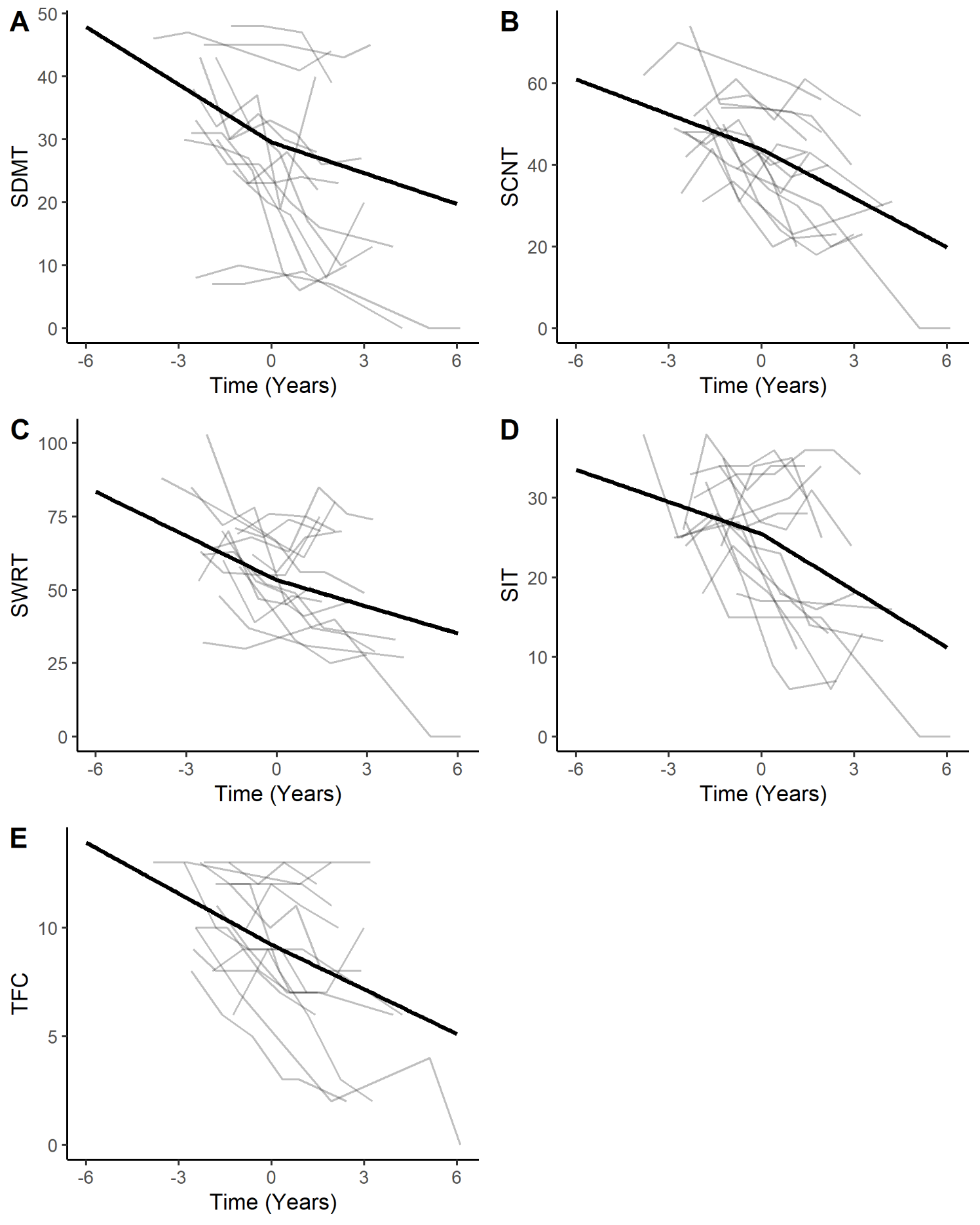
**

Time zero indicates the initiation of memantine. Values less than zero indicate assessments performed prior to the initiation of memantine and values after zero indicate assessments performed after the initiation of memantine.
